# Multiple Antibiotic-resistant Bacteria on Fluted Pumpkin Leaves, a Herb of Therapeutic Value

**Published:** 2014-06

**Authors:** Oluwatoyin A. Igbeneghu, Abdulrasheed B. Abdu

**Affiliations:** ^1^Department of Pharmaceutics, Faculty of Pharmacy, Obafemi Awolowo University, Ile-Ife, Osun State, Nigeria; ^2^Department of Medical Microbiology and Parasitology, Faculty of Basic Medical Sciences, College of Health Sciences, Niger Delta University, Wilberforce Island, Bayelsa-State, Nigeria

**Keywords:** Antianaemic, Antimicrobial resistance, Epiphytic bacteria, Fluted pumpkin, Green leafy vegetable, Pathogens, Nigeria

## Abstract

Fluted pumpkin *(Telfairia occidentalis)* is a minimally-processed green leafy vegetable traditionally used for its antianaemic properties in the form of leaf juice without a heating or inactivation step before consumption. The aim of the study was to assess the presence of surface microbiota on *T. occidentalis* leaves and also to determine the antimicrobial susceptibility of isolated organisms. Bacterial contaminants on 50 samples of *T. occidentalis* leaves were isolated and characterized using standard biochemical methods and the antimicrobial susceptibility of isolated organisms was determined using the antibiotic disc diffusion assay. The results obtained show that the leaves of *T. occidentalis* is contaminated with organisms which included *Enterobacter agglomerans* (25.9%), *Proteus vulgaris* (24.9%), *Klebsiella* spp. (2.6%), and *Serratia liquefaciens* (2.1%). Other bacterial isolates recovered in order of frequency included: *Staphylococcus* spp. (33.7%), *Bacillus* spp. (8.3%), and *Pseudomonas fluorescens* (2.6%). Of the 193 bacterial isolates from the leaves of *T. occidentalis* samples tested for antimicrobial resistance, all (100%) were found to be resistant to ampicillin, cloxacillin, augmentin, erythromycin, and tetracycline while 96% of the isolates were resistant to cephalothin. Resistance to trimethoprim (93%) and gentamicin (83%) was also observed. Approximately, 22% of the isolates were resistant to ciprofloxacin; however, only 11 (5.8%) were resistant to ofloxacin. Thus, uncooked *T. occidentalis* is a potential source of highly-resistant epiphytic bacteria which could be opportunistic pathogens in consumers.

## INTRODUCTION

*Telfairia occidentalis* Hook F. (Family: Cucurbitaceae), commonly called fluted pumpkin or fluted gourd, is a tropical vine grown in the forest zone of West and Central Africa, particularly in Benin Republic, Cameroon, Nigeria, and Sierra Leone, for its edible seeds and leaves (1). In Nigeria, the herbal preparation of the plant has been employed in the treatment of sudden attacks of convulsion, malaria, and anaemia (1). Its action against hypercholestolaemia has been investigated and reported (2-3). The plant has also been reported to possess other medicinal properties which include antimalarial (4), antidiabetic (5-6), antioxidant, and antimicrobial (7) activities. Scientific evidence supporting its use as an antianaemic medicine has also been documented (8).

Juices prepared from fluted pumpkin leaves have been claimed to increase the haemoglobin levels rapidly in the human body during the treatment of anaemia. A study reported that dietary intake of the leaf could prevent garlic-induced haemolytic anaemia in rats (9). These studies provide the scientific justification for the common practice among pregnant women in Nigeria who take the leaf juice on a regular basis. The preparation of the juice of fluted pumpkin leaves for this purpose involves picking the leaves from the tendrils, washing the leaves in water, and then squeezing or rubbing the leaves in some quantity of water to get out the leaf extract. Some other consumers mill the leaves in a blender and sieve the resulting slurry to obtain the juice. The juice is either taken immediately or stored in the refrigerator to avoid spoilage of the juice. These procedures do not involve any heating or inactivation step before consumption. This kind of preparation is prone to an increased risk of exposure to contaminants, including human pathogens (10-11). Even though spoilage bacteria, yeasts, and moulds dominate the microflora on raw fruits and vegetables, the occasional presence of pathogenic bacteria, parasites, and viruses capable of causing human infections has been documented (12-14). Contamination of the leaves may take place at all stages of production (both pre- and postharvest), and possible sources are soil, manure (of human and animal faecal origin), water (irrigation and cleaning), animals (including insects and birds), handling of the equipment for harvesting products and processing, and the mode of transportation (15-16). Very little information is documented on the occurrence of pathogens and non-pathogens on the leaves of fluted pumpkin (17), and data on the resistance or susceptibility of such contaminants to commonly-used antibiotics are scarce. This study was, therefore, undertaken with the aim to identify possible bacterial contaminants on the leaves and to determine their antimicrobial resistance profile to commonly-used antimicrobial agents.

## MATERIALS AND METHODS

A total of 50 samples of *T. occidentalis* were purchased from 3 different markets within Ile-Ife, a semi-urban locality in Southwestern Nigeria. The samples were packed individually in sterile polythene bags and transferred to the laboratory and tested immediately after being authenticated at the Department of Botany, Obafemi Awolowo University, Ile-Ife.

### Bacteriological procedures

#### Isolation and identification of bacterial contaminants

Contaminants on unwashed samples were isolated using a modified method as described by Osterblad *et al*. (18). Unwashed samples were analyzed so as to have a representation of all contaminants that are likely to be present in the washed leaves. For therapeutic use, the washed leaves are not expected to be free from contaminants but are expected to have much lower bacterial loads. Briefly, each sample of fluted pumpkin leaf was aseptically chopped into fine pieces, using sterile knife and board. Five gramme of each sample was aseptically weighed and transferred into 100 mL of sterile water in a conical flask, and the mixture was shaken to disperse any contaminants in the water; 0.5 mL of water, in which each leaf sample was suspended with a 1:100 dilution, was plated out on the surface of overdried nutrient agar (Oxoid, England) plates. The plates were incubated at 37 °C for 24 hours. Pure colonies with different morphological characteristics (colour, average colony-size, margin, surface elevation, opacity, and consistency of colonies) arising from each sample after incubation were transferred onto fresh plates and incubated appropriately. The isolates were subcultured onto MacConkey agar (Oxoid, England), Eosine Methylene Blue agar (Oxoid, England), Blood agar (Oxoid, England), and Mannitol Salt agar (Oxoid, England). All plates were incubated at 37 °C for 24 hours. The isolates were biochemically identified to the species level based on standard test protocols starting with Gram's stain (19-20). All Gram-positive cocci were tested for catalase production, coagulase, modified oxidase, and sugar fermentation. All Gram-negative isolates were tested for citrate utilization, indole production, urease production, oxidase and hydrogen sulphide production, acid and/or gas production in Triple Sugar Iron agar. The identity of each Gram-negative isolate was confirmed to species level, using API 20E (Biomérieux, France).

#### Antibiotic susceptibility tests

The susceptibility of the isolates to antibiotics was determined using the disc diffusion method approved by the Clinical Laboratory Standards Institute (21); 2-3 distinct colonies of each test organism taken from a nutrient agar culture was inoculated into 5 mL of sterile water, using a sterile loop. The suspension was thoroughly mixed with a spin mixer and adjusted to 10^6^, using 0.5 McFarland Standard as a guide. The resulting suspension was applied to the surface of overdried Mueller Hinton agar and spread evenly with a sterile cotton-tipped applicator (Sterilin Ltd, Middlesex, UK). The inoculated plates were incubated at 37 °C for 20 minutes for acclimatization and growth of the inoculum. Antibiotic discs were then lightly but firmly pressed onto the surface of the plates, using sterile forceps and placed equidistant to each other. The plates were refrigerated after application of the discs at 4 °C for 30 minutes to ensure adequate diffusion of antibiotics. The test was carried out in duplicate, and *E. coli* (ATCC 25922) was used as control Gram-negative organism while *S. aureus* (ATCC 25923) was used as the Gram-positive control. All plates were incubated at 37 °C for 18 hours. The diameters of inhibition zones were measured in mm and interpreted in accordance with the manufacturer's recommendations. The antibiotic tested included: ampicillin (10 µg), augmentin (30 µg), cloxacillin (30 µg), cephalothin (30 µg), ciprofloxacin (5 µg), chloramphenicol (30 µg), trimethoprim (5 µg), gentamicin (10 µg), tetracycline (30 µg), ofloxacin (5 µg), and erythromycin (30 µg) (Remel, USA). The organisms were categorized as resistant or susceptible based on the recommendation of the antibiotic disc manufacturers.

## RESULTS

A total of 193 bacterial isolates were recovered from all the 50 samples of *T. occidentalis* leaves analyzed. The isolates comprised 112 (58%) Gram-negative organisms which included *Proteus vulgaris*, *Enterobacter* spp., *Klebsiella* spp., *Serratia liquefaciens*, and *Pseudomonas fluorescens*. The other 81 (42%) bacterial isolates were Gram-positive organisms which included *Staphylococcus aureus* and *Bacillus cereus* (Table 1). As shown in Table 2, the contaminants were found to co-occur on the leaves with samples bearing 2-3 contaminants in the majority. The results obtained on the antimicrobial susceptibility test showed that all the samples carried antibiotic-resistant bacterial contaminants, with 7 of the samples (14%) found to be contaminated with organisms that were resistant to all antibiotics tested. As illustrated in the figure, the isolates were resistant to a wide range of antimicrobial agents, particularly the penicillins (100%) and tetracycline (100%). These were, however, susceptible to the quinolones. The distribution of antibiotic resistance among the isolated bacterial species is shown in Table 3.

## DISCUSSION

Recent outbreaks of infectious diseases associated with fruits and vegetables have prompted increased interest in all aspects of the microbiologic safety of fruits and vegetables, particularly those that are eaten raw or subjected to minimal processing only prior to their consumption (22-23).

The result of this study showed that, as in many plants, a microbial flora exists on the leaves of *T. occidentalis*. The flora comprised both Gram-positive and Gram-negative bacteria, with the latter group of organisms in the majority. These organisms are probably contaminants introduced right on the farm during cultivation and harvesting, from the soil, farm implements, and the farmers (15-16). Some of the clinically-significant Gram-positive organisms isolated in this study were: *Staphylococcus aureus*, *Staphylococcus epidermidis*, *Bacillus cereus,* and *Bacillus subtilis*. The *Bacillus* spp. were isolated from about a quarter of the samples tested. This pattern of isolation corroborates an earlier report implicating *Bacillus* spp. as one of the common contaminants of vegetable materials (24). Similarly, the organisms were isolated from samples of *T. occidentalis* from a different (south-southern) region of Nigeria (17). These are soil-originated organisms that have the ability to persist on harvested farm produce right from the farm to the table because of their ability to form spores. In a case of minimally-processed fluted pumpkin leaves for its juice, *Bacillus cereus* and *subtilis* may pose some health hazards if ingested in sufficient quantity to cause a poisoning as the case may be when the juice is not consumed immediately after preparation. *Staphylococcus* spp. were isolated from over 80% of the samples in this study, with *Staphylococcus aureus* making up about 30%. Unlike *Bacillus* spp., *Staphylococcus aureus* and *Staphylococcus epidermidis* are more likely to have been introduced onto the leaves from the hands of the handlers which include the farmers and the sellers. As ascribed by Madden (25), this could be from the point of harvesting to the packaging, sizing for sale, and also from the utensils and packaging bags used by the sellers. The ingestion of a sufficient load of *Staphylococcus aureus* is also undesirable due to the ability of *Staphylococcus aureus* to cause serious food poisoning with fatal consequences.

**Table 1.** Organisms isolated from *T. occidentalis* leaves

Organism	No. isolated (%)	No. of samples contaminated (%)
*Proteus vulgaris*	48 (24.9)	33 (66)
*Pantoea agglomerans*	35 (18.1)	35 (70)
*Staphylococcus aureus*	19 (9.8)	19 (38)
*Enterobacter aerogenes*	12 (6.2)	12 (24)
*Staphylococcus epidermidis*	11 (5.7)	11 (22)
*Bacillus subtilis*	8 (4.2)	6 (12)
*Bacillus cereus*	6 (3.1)	5 (10)
*Pseudomonas fluorescens*	5 (2.6)	3 (6)
*Serratia liquefaciens*	4 (2.1)	4 (8)
*Klebsiella pneumoniae*	4 (2.1)	4 (8)
*Enterobacter cloacae*	3 (1.6)	3 (6)
*Bacillus sphearicus*	2 (1.0)	2 (4)
*Klebsiella oxytoca*	1 (0.5)	1 (2)
Unspeciated coagulase-negative staphylococci	35 (18.1)	25 (50)
Total	193	

**Table 2.** Multiple contaminants profile of samples

No. of contaminants	No. of samples	% of sample
1	4	8
2	24	48
3	12	24
4	8	16
5	2	8
Total	50	100

**Table 3.** Distribution of antibiotic resistance among isolated bacterial species

Organism (number isolated)	No. of isolates resistant to antibiotics (%)					
	Ceph	Chlo	Gent	Trim	Cip	Ofl
*Pr. vulgaris* (48)	45 (94)	48 (100)	48 (100)	48 (100)	5 (10)	5 (10)
*P. agglomerans* (35)	35 (100)	35 (100)	34 (97)	35 (100)	4 (11)	4 (11)
*S. aureus* (19)	19 (100)	16 (84)	9 (47)	19 (100)	8 (42)	10 (53)
*E. aerogenes* (12)	12 (100)	12 (100)	12 (100)	10 (83)	1 (8)	1 (8)
*S. epidermidis* (11)	10 (91)	5 (46)	0 (0)	6 (55)	0 (0)	0 (0)
*B. subtilis* (8)	5 (63)	5 (63)	5 (63)	8 (100)	2 (25)	2 (25)
*B. cereus* (6)	2 (33)	6 (100)	2 (33)	6 (100)	1 (17)	1 (17)
*P. fluorescens* (5)	5 (100)	5 (100)	5 (100)	5 (100)	5 (100)	5 (100)
*S. liquefaciens* (4)	4 (100)	4 (100)	4 (100)	4 (100)	4 (100)	4 (100)
*K. pneumonia* (4)	4 (100)	4 (100)	4 (100)	4 (100)	2 (50)	2 (50)
*E. cloacae* (3)	3 (100)	3 (100)	3 (100)	3 (100)	1 (33)	1 (33)
*B. sphearicus* (2)	2 (100)	1 (50)	1 (50)	1 (50)	0 (0)	0 (0)
*K. oxytoca* (1)	1 (100)	1 (100)	0 (0)	1 (100)	0 (0)	0 (0)
Unspeciated CoNS (35)	35 (100)	35 (100)	35 (100)	30 (86)	12 (34)	16 (46)

Ceph=Cephalothin, Chlo=Chloramphenicol, Gent=Gentamicin, Trim=Trimethoprim, Cip=Ciprofloxacin, and Ofl=Ofloxacin; CoNS=Coagulase-negative staphylococci

**Figure.**
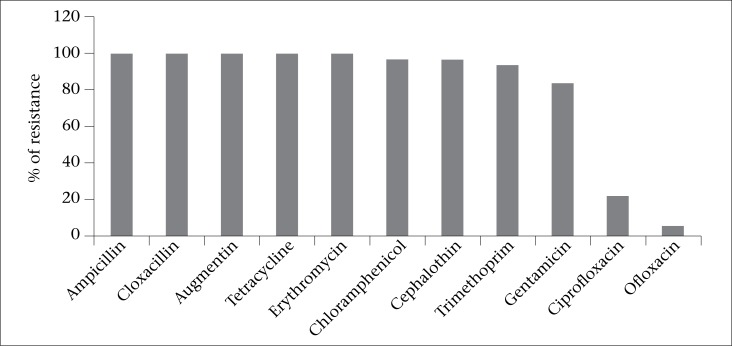
Antimicrobial resistance profile of *T. occidentalis* leaf contaminants

The Gram-negative organisms isolated in this study were predominantly of the family Enterobacteriaceae. The absence of *E. coli* indicated the absence of recent human or animal faecal contaminants on the leaves. The isolated Enterobacteriaceae could have come from the soil and the contaminated water used in irrigation and washing of the vegetables. The presence of some Enterobacteriaceae on some vegetable samples in Nigeria has been shown to be related to the water source employed in the irrigation of the vegetables during cultivation (26). To maintain the freshness of the leaves, the sellers usually sprinkle water at intervals on fluted pumpkin leaves when displayed for sale. This practice as documented by various authors could, however, provide a moist surface that encourages the growth of members of the family Enterobacteriaceae (27-28).

The genus *Enterobacter* which was recovered from 84% of the samples in this study was nominated as ‘emerging pathogen of the millenium’ (29) and is regarded as medically significant. On the other hand, *Pantoea agglomerans* isolated from our samples has been reported as a cause of opportunistic infections (30). A study similarly reported the isolation of *Enterobacter aerogenes* from *T. occidentalis* leaves in Calabar, Cross-River State of Nigeria (17) while another reported its association with vegetables in the UK (31). *Proteus vulgaris* and *Klebsiella* species have similarly been associated with various vegetables (17); and *Klebsiella* spp., along with *Acinetobacter* spp., was isolated from mustard and cress in green salad prepared in a hospital kitchen (32).

The Gram-negative organisms other than those of the family Enterobacteriaceae isolated in this study were strains of *Pseudomonas fluorescens*, which are reported as common inhabitants of plant surfaces (33) and are implicated as causative agents of opportunistic and nosocomial infections (34-35).

In a larger proportion of the samples (72%), 2 to 3 of the isolated organisms were found as co-contaminants on the leaves. Some (24%) had 4-5 contaminants at a time. The health implication of this is that it could further compound the problem of infection from unprocessed juice; as such, infections are going to be mixed infections, thus posing difficulties in identifying and treating an infection that could arise from the ingestion of such leaves. In cases where the microbial load on harvested leaves has been adequately reduced using potable water, the presence of just a few cells of the organisms is still significant, especially in the immune-compromised persons in whom these cause a health hazard.

Much attention has been paid to the role animal-derived foods play in the acquisition and persistence of antibiotic resistance in humans (36), and it is assumed that the acquisition of antimicrobial resistance from these foods will adversely affect the efficacy of clinical antimicrobial chemotherapy (37). To date, foods of plant origin have received scant attention in this regard. From this study, the Gram-negative organisms isolated from the leaves of *T. occidentalis* were found to be resistant to a wide range of antimicrobials used in the clinical settings within our study environments. Our findings showed that most of the isolates were resistant to multiple antibiotics. Similar multidrug-resistant epiphytic bacteria have been reported in Finland (18) and in the United Kingdom (31).

All our isolates were resistant to the action of the beta-lactam antibiotics as has been observed in other studies of food plant-associated Gram-negative bacteria (31,38). Screening of Gram-negative bacteria recovered from fresh and salad vegetables obtained at retail outlets in Germany, Finland, and the United States revealed high incidence of resistance (70–90%) to the beta-lactam antibiotics, ampicillin, and cephalothin (31,38-39). Levels of resistance were equally high (83.4-100%) among these Nigerian isolates to the other antimicrobial compounds (e.g. aminoglycosides, tetracycline, and chloramphenicol), which is contrary to reports from Germany, Finland, the UK, and the United States. In these countries, such levels of resistance were less frequent and ranged from 4 to 43% depending on the particular antibiotic (31,38-39). These high rates of resistance are usually not characteristic of the environmental isolates. The observation, thus, suggests that these isolates could be of animal or human origin in which overuse or abuse of antibiotics create a selective pressure for antibiotic-resistant organisms. The use of animal manure in the cultivation of fluted pumpkin may be associated with the observed resistance. Symptomatic infections caused by these multidrug-resistant organisms may be very difficult to treat as almost all the available and cheap antimicrobial agents will be ineffective. Antimicrobial drugs of choice may be limited to the quinolones. Of more concern is the fact that these Enterobacteriaceae are the most notorious organisms in nature known to contribute significantly to the spread of antibiotic resistance in the community through conjugative plasmids (40).

### Conclusions

This study has shown that the high levels of resistance in humans in this environment are not only due to the abuse or indiscriminate use of antibiotics in humans but also due to the presence of resistant organisms in herbs or foods eaten raw or minimally processed, such as *T. occidentalis* leaf juice. To curtail the rising incidences of resistance in this environment, measures to control the microbial contamination of foods and herbs, especially those eaten without any processing methods that kill contaminants, must be put in place. The elimination of contaminants, strict adherence to good manufacturing practices, and sanitation will be required in the production or preparation of *T. occidentalis* juice for therapeutic use in humans.
